# High Consumption of Iron Exacerbates Hyperlipidemia, Atherosclerosis, and Female Sterility in Zebrafish via Acceleration of Glycation and Degradation of Serum Lipoproteins

**DOI:** 10.3390/nu9070690

**Published:** 2017-07-02

**Authors:** So-Hee Kim, Dhananjay Yadav, Suk-Jeong Kim, Jae-Ryong Kim, Kyung-Hyun Cho

**Affiliations:** 1 Department of Medical Biotechnology, Yeungnam University, Gyeongsan 712-749, Korea; ddccom@net.com (S.-H.K.); dhanyadav16481@gmail.com (D.Y.); superiorgene@ynu.ac.kr (S.J.K.); 2 Research Institute of Protein Sensor, Yeungnam University, Gyeongsan 712-749, Korea; 3 BK21plus Program Serum Biomedical Research and Education Team, Yeungnam University, Gyeongsan 712-749, Korea; 4 Department of Biochemistry and Molecular Biology, College of Medicine, Yeungnam University, Daegu 705-717, Korea; kimjr000@gmail.com

**Keywords:** iron, lipoprotein, hyperlipidemia, cellular senescence, macrophages, human dermal fibroblast cells

## Abstract

Elevated serum iron level is linked with an increased risk of diabetes and atherosclerosis. However, the pathological mechanism by which iron affects serum lipoprotein levels is unknown. To elucidate the mechanism, a high dose of ferrous ion was applied (final 60 µM, 120 µM) to human serum lipoproteins, macrophages, and human dermal fibroblast (HDF) cells. Iron-treated lipoproteins showed loss of antioxidant ability along with protein degradation and multimerization, especially co-treatment with fructose (final 10 mM). In the presence of fructose, HDF cells showed 3.5-fold more severe cellular senescence, as compared to the control, dependent on the dosage of fructose. In macrophages, phagocytosis of acetylated low-density lipoprotein (acLDL) was more accelerated by ferrous ion, occurring at a rate that was up to 1.8-fold higher, than acLDL alone. After 24 weeks supplementation with 0.05% and 0.1% ferrous ion in the diet (wt/wt), serum total cholesterol (TC) level was elevated 3.7- and 2.1-fold, respectively, under normal diet (ND). Serum triglyceride (TG) was elevated 1.4- and 1.7-fold, respectively, under ND upon 0.05% and 0.1% ferrous ion supplementation. Serum glucose level was elevated 2.4- and 1.2-fold under ND and high cholesterol diet (HCD), respectively. However, body weight was decreased by the Fe^2+^ consumption. Iron consumption caused severe reduction of embryo laying and reproduction ability, especially in female zebrafish via impairment of follicular development. In conclusion, ferrous ion treatment caused more pro-atherogenic, and pro-senescence processes in human macrophages and dermal cells. High consumption of iron exacerbated hyperlipidemia and hyperglycemia as well as induced fatty liver changes and sterility along with reduction of female fertility.

## 1. Introduction

Iron is an essential nutrient and component of hemoglobin, which binds to oxygen in the heme structure, as well as the mitochondrial electron transport system. Although iron exists in trace amounts in the body, its role is very critical in many metabolic processes, including electron transport and respiration [1]. Iron serves as a cofactor for several enzymes involved in oxidation and reduction processes due to its ability to exist in two ionic forms, ferrous (Fe^2+^) and ferric (Fe^3+^) ions [2]. Iron is an essential nutrient for life functions, including red blood cell function, oxygen transfer, DNA synthesis, protein synthesis, hormone synthesis, and cell replication [3]. Iron deficiency is frequently associated with anemia, neurodegenerative disease, and retardation of growth along with behavioral disorders, especially in children [4].

However, an excess amount of iron can be dangerous, since it is a potent oxidizing agent and can damage tissue by catalyzing the conversion of hydrogen peroxide into free radical ions via the Fenton reaction (Fe^2+^ + H_2_O_2_ → Fe^3+^ + OH^−^ + HO) [5]. It is well known that free hydroxyl radicals are associated with aging stress, autoimmunity, bone loss, and cancer development [6]. Since iron storage and excretion capacities in human organs are limited, daily consumption of iron-enriched foods can cause an overload of iron in tissues, resulting in the production of highly reactive iron complexes [7].

It is also well known that free radicals can oxidize low-density lipoproteins (LDLs) in endothelial cells, smooth muscle cells, lymphocytes, and macrophages [8]. Oxidized LDL (oxLDL) is easily taken up into macrophages for conversion of foam cells and progression of fatty streak lesions [9]. It has been known that high consumption of iron-enriched foods can result in excessive iron accumulation, interfere with glucose disposal from the liver, and decrease insulin secretion from pancreatic β-cells [2,10]. Excessive iron can also exacerbate metabolic diseases related to glucose and lipid dysfunction such as diabetes and cardiovascular disease, respectively [11]. Ferrous ion (Fe^2+^) can also induce mild oxidation, and iron in heme group can cause strong oxidation of LDL. Modification of LDL by hemoglobin is known to result in crosslinking between apo-B and Hb-apo-B [12]. However, there has been no report about the influence of ferrous ion on HDL metabolism.

It has been well reported that high-density lipoprotein (HDL) is inversely related to the incidence of coronary heart disease. HDL is an apolipoprotein-lipid complex in plasma that exerts potent antioxidant, anti-inflammatory, and anti-atherosclerotic effects [13]. ApoA-I, the major protein component of HDL, has several beneficial activities for maintaining healthy HDL function. The quality of HDL is highly dependent on the structural and functional correlations of apoA-I during aging and disease processes [14]. Our group reported that modification of apoA-I is directly associated with production of dysfunctional HDL [15], which has more atherogenic and inflammatory properties that exacerbate senescence and aging-related diseases.

Although the physiological effect of excess iron on lipoprotein metabolism is still unknown, iron taken from the intestine can be transported into the bloodstream. In blood, a putative interaction can occur between lipoproteins and iron in the form of ferrous or ferric ion. It is plausible that LDL and HDL can be easily oxidized and modified to produce more atherogenic proteins by ferrous ion. However, the physiological effects of modified lipoproteins by iron, especially detailed pathological mechanisms, have not been determined in the context of lipid and lipoprotein metabolism.

In order to investigate the physiological effects of high iron consumption on lipoprotein metabolism, ferrous ion was administered to human LDL and HDL, macrophages and human dermal fibroblast (HDF) cells, and hyperlipidemic zebrafish.

## 2. Materials and Methods

### 2.1. Chemicals

Ferrous ion (Iron (II) sulfate heptahydrate, cat No. 215422) was purchased from Sigma (St. Louis, MO, USA).

### 2.2. Purification of Human Lipoproteins and ApoA-I

Human plasma was isolated by low-speed centrifugation from healthy human males (mean age, 22 ± 2 years), who fasted for at least 16 h before donating blood voluntarily. Very-low-density lipoprotein (VLDL, *d* < 1.019 g/mL), low-density lipoprotein (LDL, 1.019 < *d* < 1.063), high-density lipoprotein (HDL_2_, 1.063 < *d* < 1.125), and HDL_3_ (1.125 < *d* < 1.225) were isolated via sequential ultracentrifugation, and the density was appropriately adjusted by the addition of NaCl and NaBr in accordance with standard protocols [16]. Samples of each lipoprotein fraction were centrifuged for 22 h at 10 °C and 100,000 *g* using a Himac CP-90α (Hitachi, Tokyo, Japan) at the Instrumental Analysis Center at Yeungnam University. After centrifugation, each lipoprotein sample was extensively dialyzed against Tris-buffered saline (TBS; 10 mM Tris-HCl, 5 mM ethylenediaminetetraacetic acid (EDTA), and 140 mM NaCl (pH 7.4)) for 24 h in order to remove NaBr.

Human apoA-I was purified from HDL by ultracentrifugation and column chromatography according to a previously described method [17]. Protein purity of at least 95% was confirmed by sodium dodecyl sulfate polyacrylamide gel electrophoresis (SDS-PAGE). Protein concentration was determined according to Lowry-Markwell protein assay, which was modified for lipoproteins as in our previous report [18], using bovine serum albumin as a standard.

### 2.3. Treatment of Lipoproteins with Ferrous Ion

Ferrous ion was individually administered to purified HDL_2_ and HDL_3_ (1 mg of protein), followed by incubation for the designated times from 24 h to 96 h at 37 °C in the presence of 5% CO_2_. In order to maintain consistency with in vivo dosages (5 and 10 µg per 300 mg of zebrafish body weight), we administered final concentrations of 60 and 120 µM FeSO_4_. After incubation, lipoproteins were analyzed by electrophoresis (SDS-PAGE) and fluorospectroscopy in order to confirm the extent of glycation.

### 2.4. Acceleration of LDL Oxidation

In order to determine the extent of oxidation, purified human LDL was incubated with ferrous ion (final 60, 120 µM) in the presence of 10 µM CuSO_4_ (final conc). After 6 h, oxidized samples were subjected to electrophoresis on 0.5% agarose gels to compare electrophoretic mobility [19]; migration of each lipoprotein is known to depend on its intact charge and size. Gels were then dried and bands stained with 1.25% Coomassie Brilliant Blue.

### 2.5. Western Blotting

In order to identify the modification of HDL_3_ by iron, ferrous ion (final 60, 120 µM) was treated to purified HDL_3_ (1 mg of protein), followed by incubation for the designated times at 37 °C in the presence of 5% CO_2_. After 96 h of incubation, about 2 µg of protein was loaded and electrophoresed on 15% SDS-PAGE gels and detected using anti-human full-length apoA-I goat antibody (ab7613; Abcam, Cambridge, UK) and donkey anti-goat IgG-HRP (ab6885, Abcam, Cambridge, UK) as secondary antibody (1:4000 diluted).

### 2.6. LDL Phagocytosis Assay

THP-1 cells, a human monocytic cell line, were obtained from the American Type Culture Collection (ATCC, #TIB-202™, Manassas, VA, USA) and maintained in RPMI-1640 medium (Hyclone, Logan, UT, USA) supplemented with 10% fetal bovine serum (FBS) until experimentation. Cells that had undergone no more than 20 passages were incubated in medium containing phorbol 12-myristate 13-acetate (PMA; final concentration, 150 nM) in 24-well plates for 24 h at 37 °C in a humidified incubator (5% CO_2_, 95% air) to induce differentiation into macrophages. Differentiated and adherent macrophages were then rinsed with warm phosphate buffered saline (PBS) and incubated with 400 µL of fresh RPMI-1640 medium containing 1% FBS, 50 µL of acetylated low-density lipoprotein (acLDL) (1 mg of protein/mL in PBS), and 50 µL of PBS or ferrous ion (final 60, 120 µM of ferrous ion in ddH_2_O) for 48 h at 37 °C in a humidified incubator. After incubation, cells were washed with PBS three times and then fixed in 4% para-formaldehyde for 10 min. Next, fixed cells were stained with oil-red O staining solution (0.67%) and then washed with distilled water. THP-1 macrophage-derived foam cells were then observed and photographed using a Nikon Eclipse TE2000 microscope (Tokyo, Japan) at 600× magnification.

### 2.7. Cellular Senescence Assay

Human dermal fibroblasts (HDFs) were cultured in Dulbecco’s modified Eagle’s medium (DMEM; Life Technologies, Gaithersburg, MD, USA). HDFs were plated in DMEM at a density of 1 × 10^5^ cells per 100-mm culture plate and cultured at 37 °C in a 5% CO_2_ humidified incubator as described in our previous report [20]. HDFs were exposed at passage 11 (approximately 40% confluence) to the indicated concentrations of ferrous ion (60, 120 µM) for 30 days with subculture to passage 18. The extent of aging and cellular SA-β-gal activity were compared as previously described [21] based on intensity and extent of staining.

### 2.8. Zebrafish

Wild-type zebrafish (Linebrass, AB strain) and embryos were maintained according to standard protocol [22]. Maintenance and experimental procedures for zebrafish were approved (YUHS 01-13-004) by the Committee of Animal Care and Use of Yeungnam University (Gyeongsan, Korea). Fish were maintained in a system cage at 28 °C during treatment under a 14:10 h light:dark cycle.

### 2.9. Supplementation of Ferrous Ion to Zebrafish

Tetrabit (47.5% crude protein; 605% crude fat; 2.0% crude fiber; 10.5% crude ash; vitamin A, 29770 IU/kg; vitamin E, 200 mg/kg; and vitamin C, 137 mg/kg; Tetrabit Gmbh D49304, Melle, Germany) was mixed with ferrous ion (final concentrations, 0.05% and 0.1%. respectively, in tetrabit (wt/wt)). The two dosages of ferrous ions in the diet were selected based on suggestions of previous reports in a murine model [23,24]. After dissolving completely in water, each mixture was lyophilized and ground into powder. Tetrabit alone or containing ferrous ion was further mixed with cholesterol to make a high cholesterol diet (HCD). A HCD containing 4% cholesterol (final concentration) was made by soaking tetrabit in a diethyl ether solution of cholesterol, as previously described [25].

As designated in Table 1, blood (2 µL) was drawn from the hearts of adult fish after feeding for 24 weeks, combined with 5 µL of PBS-EDTA, and then collected into EDTA-treated tubes (final 1 mM). Serum total cholesterol (TC), HDL-cholesterol, and triglyceride (TG) levels were determined using a commercial assay kit (cholesterol, T-CHO; Wako Pure Chemical, Osaka, Japan; triglycerides, Cleantech TS-S, Wako Pure Chemical). Concentrations of aspartate aminotransferase (AST) and alanine aminotransferase (ALT) were measured using a commercially available assay kit (Asan Pharmaceutical, Hwaseong, Korea).

### 2.10. Histologic Analysis

Testes and ovaries were collected from zebrafish fed a normal diet (ND) or HCD for 24 weeks. Tissues were fixed in Bouin solution for 2 days and then dehydrated in 30% sucrose solution for 2 days. Frozen testes in optimum cutting temperature (OCT) cryo-embedding compound were freshly sectioned to a thickness of 6 µm and stained using standard hematoxylin and eosin (H & E) staining protocol. H & E staining was also performed on formalin-fixed, paraffin-embedded 3-μm sections after deparaffinization, as previously described [26]. Testicular abnormalities were examined by measuring spermatogenesis, size of seminiferous tubules, as well as size of the interstitial space in the testis slides from at least three fishes per group. Spermatogenic defects were examined by measuring the area of each spermatogenic cell, as previously defined by [27]. Ovaries were collected from zebrafish fed an iron-enriched diet (0.05% and 0.1% ferrous ion) with or without HCD for 24 weeks. Tissues were fixed in Bouin solution for 2 days and then dehydrated in 30% sucrose solution for 2 days. Frozen ovaries in OCT cryo-embedding compound were freshly sectioned to a thickness of 4 µm and stained using standard H & E staining protocol. Ovary abnormalities were examined by measuring folliculogenesis according to Selman’s classification [28].

### 2.11. Statistical Analysis

All data are expressed as the mean ± SD from at least three independent experiments with duplicate samples. Comparisons between results were made by Student’s *t*-test using the SPSS program (version 12.0; SPSS, Inc., Chicago, IL, USA). In the zebrafish study, data in the same group were evaluated via one-way analysis of variance (ANOVA) using SPSS (version 14.0; Chicago, IL, USA), and differences between the means were assessed using Duncan’s multiple-range test. Statistical significance was defined as a *p* < 0.05.

## 3. Results

### 3.1. Lipoprotein Modification by the FeSO_4_

**Figure 1:**
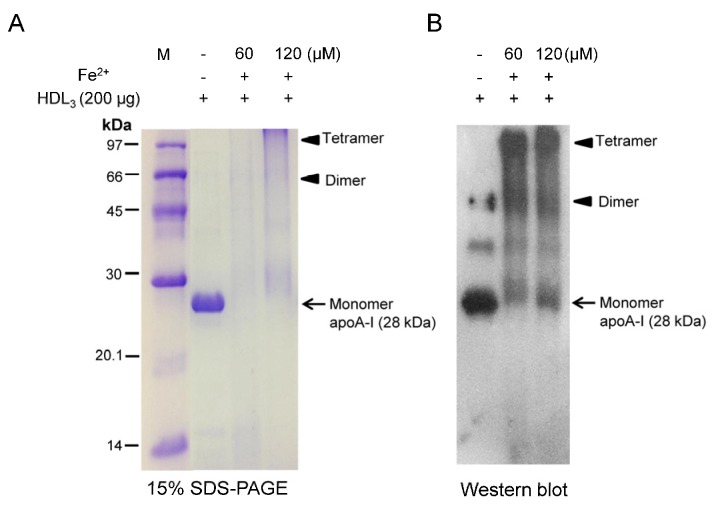
Electrophoretic patterns of human high density lipoprotein (HDL_3_) after Fe^2+^ treatment for 96 h. (**A**) Proteolytic degradation of apoA-I in HDL_3_ upon Fe^2+^ treatment (15% sodium dodecyl sulfate polyacrylamide gel electrophoresis (SDS-PAGE)). Protein bands were visualized by Coomassie brilliant blue R-250; (**B**) Immunodetection of apoA-I in HDL_3_ with apoA-I antibody (ab7613, Abcam). Aggregated and multimerized apoA-I bands were detected up to tetramer.

FeSO_4_-treated HDL_3_ showed severe proteolysis after 96 h of incubation with two dosages (final 60 and 120 µM) at 37 °C in the presence of 5% CO_2_, as shown in Figure 1. ApoA-I band (28 kDa) disappeared upon 60 µM FeSO_4_ treatment and showed more smearing upon 120 µM FeSO_4_ treatment, as shown in Figure 1A. To confirm degradation of apoA-I, western blot analysis revealed multimerization of apoA-I while the 28 kDa band was shifted up slightly (as indicated arrow) upon FeSO_4_ treatment in a dose-dependent manner, as indicated by the arrowhead (Figure 1B).

### 3.2. Acceleration of HDL Glycation by FeSO_4_

**Figure 2:**
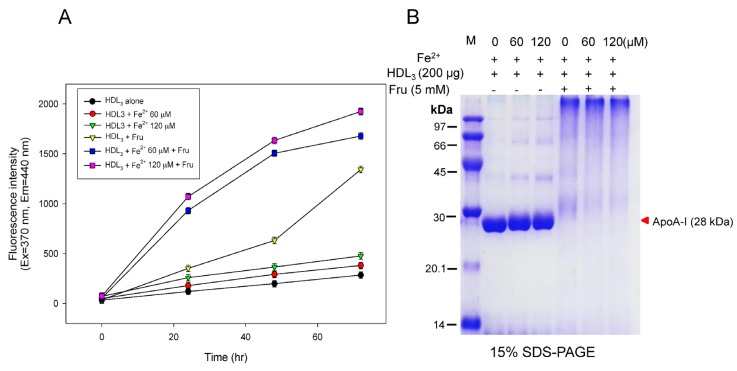
Modification of HDL by Fe^2+^ treatment with or without fructose for induction of glycation. (**A**) Production of glycated end products based on detection of fluorescence (Ex = 370 nm, Em = 440 nm); (**B**) Electrophoretic patterns of Fe^2+^-treated HDL after 72 h of incubation depends on ferrous ion dosage.

Fructation of HDL_3_ was more accelerated in the presence of FeSO_4_ during 72 h of incubation in a dose-dependent manner, as shown in Figure 2A. Although the glycation extent increased around 1.3-fold without fructose, glycation was elevated 4.7-fold upon co-treatment with fructose. Especially, co-treatment with fructose and FeSO_4_ (final 120 µM) caused a 6.7-fold increase compared to HDL_3_ alone, suggesting that there is a synergistic effect of glycation between fructose and FeSO_4_ for induction of modification.

In order to confirm glycation, SDS-PAGE analysis revealed that FeSO_4_ treatment alone caused slight smearing of the apoA-I band along with multimerization (Figure 2B). However, co-treatment with fructose and FeSO_4_ caused severe proteolytic degradation of the apoA-I band (28 kDa) along with aggregation.

### 3.3. Synergistic Effect of ApoA-I Glycation by FeSO_4_ and Fructose

**Figure 3:**
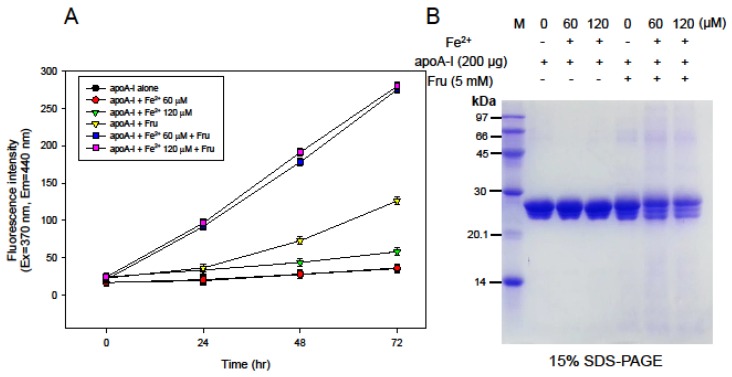
Treatment with Fe^2+^ to apoA-I with or without fructose for induction of glycation. (**A**) Production of glycated end products based on detection of fluorescence (Ex = 370 nm, Em = 440 nm); (**B**) Electrophoretic patterns of Fe^2+^-treated apoA-I after 72 h of incubation depends on ferrous ion dosage.

Glycation of lipid-free apoA-I was more accelerated by co-treatment with FeSO_4_ and fructose during 72 h of incubation in a dose-dependent manner (Figure 3). Glycation of apoA-I increased up to 3.6-fold compared to apoA-I alone with fructose. Furthermore, co-treatment with fructose and FeSO_4_ (final 120 µM) caused an 8-fold increase in glycation compared to apoA-I alone, suggesting a synergistic effect between fructose and FeSO_4_. However, there was no difference between the FeSO_4_ dosages. SDS-PAGE analysis revealed that FeSO_4_ treatment alone did not cause degradation of lipid-free apoA-I, as shown in Figure 3B, whereas co-treatment with fructose and FeSO_4_ caused severe proteolytic degradation of apoA-I.

### 3.4. LDL Oxidation and Phagocytosis into Macrophage Was More Facilitated by FeSO_4_

**Figure 4:**
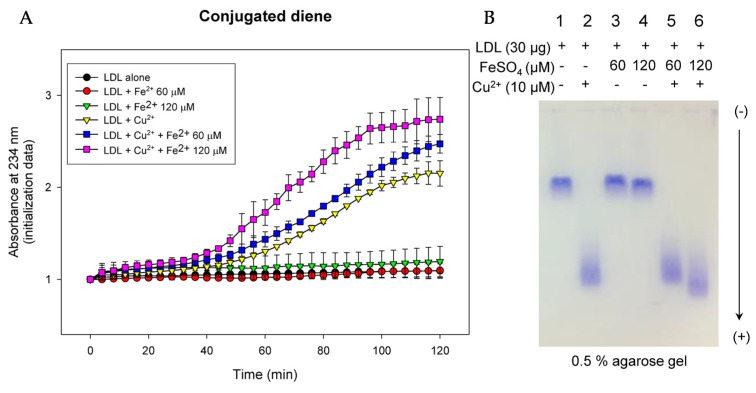
Oxidation of low-density lipoprotein (LDL) by Fe^2+^ and Cu^2+^. (**A**) Detection of conjugated diene based on absorbance at 234 nm from oxidation; (**B**) Comparison of relative electrophoretic mobility between LDL samples after Fe^2+^ and Cu^2+^-mediated oxidation. More-oxidized LDL migrates faster toward cathode in bottom of gel (0.5% agarose).

Up to 2 h of incubation, co-treatment with Fe^2+^ and Cu^2+^ (final 10 µM) caused the fastest oxidation of LDL in a Fe^2+^ dose-dependent manner, as shown in Figure 4A. Co-treatment caused 1.3-fold higher oxidation than Cu^2+^-mediated oxidation of LDL. However, Fe^2+^ treatment alone did not cause oxidation, similar with LDL alone. To confirm the spectroscopic data, electromobility of LDL was compared by agarose gel electrophoresis. Upon treatment with Cu^2+^, oxidized LDL showed the fastest electromobility (lane 2 of Figure 4B) due to an increased negative electric charge and fragmentation of apo-B. FeSO_4_ treatment accelerated oxidation in the presence of Cu^2+^ in a dose-dependent manner (lane 5 and 6, Figure 4B), whereas FeSO_4_ alone did not cause oxidation (lane 3 and 4).

**Figure 5:**
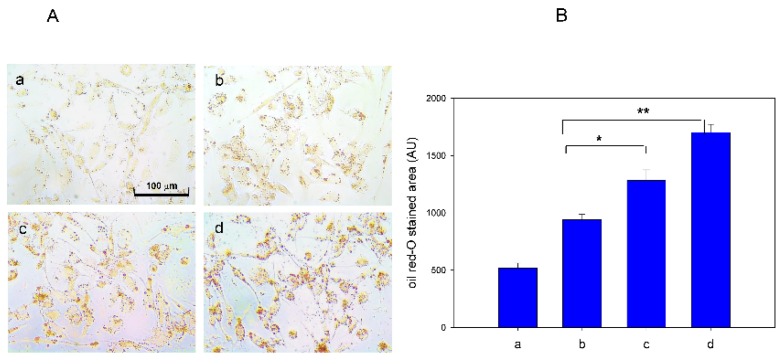
Cellular uptake of acetylated low-density lipoprotein (acLDL) in the presence of Fe^2+^. (**A**) Phorbol 12-myristate 13-acetate (PMA)-differentiated macrophages were incubated with 50 µL of acLDL (1 mg/mL), 50 µL of each Fe^2+^ solution, and 400 µL of RPMI-1640 media. Extent of cellular uptake of the acLDL by macrophages was then compared by oil red O staining, as described in the text. Cells were then photographed using a Nikon Eclipse TE2000 microscope (Tokyo, Japan) at 600× magnification. Photo a, phosphate buffered saline (PBS)-treated; Photo b, acLDL alone-treated; Photo c, acLDL + Fe^2+^ 60 µM (final); Photo d, acLDL + Fe^2+^ 120 µM; (**B**) Quantification of oil red O stained area by computer-assisted morphometry using Image Proplus software (version 4.5.1.22; Media Cybernetics, Rockville, MD, USA). AU, arbitrary unit. *, *p* < 0.05; **, *p* < 0.01.

However, as shown in Figure 5A, phagocytosis of acLDL into macrophages was more accelerated by FeSO_4_ treatment in a dose-dependent manner, as visualized by oil-red O staining. Uptake of acLDL was elevated 1.4-fold and 1.8-fold by 60 and 120 µM FeSO_4_ (final), respectively, compared with acLDL treatment alone based on red-intensity quantification (Figure 5B). Since the uptake of acLDL into macrophages is the initial atherogenic process for conversion of foam cells, excess Fe^2+^ can be an atherogenic factor in the bloodstream.

### 3.5. Cellular Senescence Was More Accelerated by FeSO_4_

**Figure 6:**
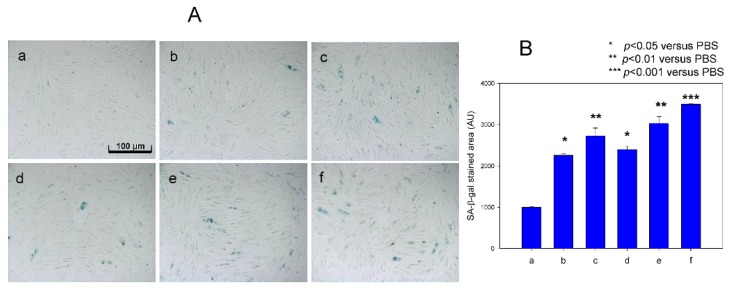
Induction of cellular senescence in human dermal fibroblasts (HDFs) under presence of Fe^2+^ by comparison of Senescence-associated β-galactosidase (SA-β-gal) staining. Cells were treated with or without fructose for 15 days (at passage = 13). (**A**) Cell image was captured using a Nikon Eclipse TE2000 microscope (Tokyo, Japan) at 600× magnification. Photo a, PBS-treated; Photo b, 60 µM Fe^2+^ (final)-treated; Photo c, 120 µM Fe^2+^ (final)-treated; Photo d, PBS + fructose (final 5 mM); photo e, fructose (final 5 mM) + 60 µM Fe^2+^ (final); photo f, fructose (final 5 mM) + 120 µM Fe^2+^ (final)-treated. (**B**) Graph shows percentage of SA-β-gal-positive cells per 7.4 mm^2^ of cell culture area during Fe^2+^ treatment to human dermal fibroblast (HDF) cells. ***, *p* < 0.001; **, *p* < 0.01; *, *p* < 0.05.

Cellular senescence of HDF cells was elevated by treatment with fructose (final 5 mM) up to 2.4-fold compared to the control (photo a), as visualized by blue intensity (photo d) in SA-β-gal assay at passage 13. However, FeSO_4_ treatment (final 60 and 120 µM) without fructose also caused senescence; 2.3- and 2.7-fold higher production of SA-β-gal positive cells (photo b and c) compared with control. In the presence of fructose, senescence was more accelerated by FeSO_4_ treatment (final 120 µM, photo f) up to 3.5-fold compared to SA-β-gal-positive cells (Figure 6), indicating that there might be a synergistic effect between FeSO_4_ and fructose for induction of cellular senescence.

### 3.6. High Iron Diet Causes Hyperlipidemia in Zebrafish

After 24 weeks of FeSO_4_ consumption (final 0.05% and 0.1% diet, wt/wt) with or without cholesterol supplementation (final 4%, wt/wt), zebrafish in normal diet (ND) and high cholesterol diet (HCD) showed significantly elevated serum total cholesterol (TC), triglyceride (TG), and glucose levels (Table 1). In the ND group, serum TC levels were elevated 3.7- and 2.1-fold compared to the control upon 0.05% and 0.1% FeSO_4_ supplementation, respectively. Under HCD, serum TC levels were elevated 1.4- and 1.3-fold by 0.05% and 0.1% FeSO_4_ consumption, respectively, suggesting no dose-dependent effect of iron. Serum TG levels increased up to 1.4- and 1.7-fold upon ND with 0.05% and 0.1% wt/wt FeSO_4_ consumption, respectively. Serum glucose level was significantly elevated up to 2.4-fold by ND and iron consumption, whereas HCD and iron consumption resulted in a 1.3-fold elevated serum glucose level. The serum AST level increased 1.5- and 2.0-fold in the ND group with 0.05% and 0.1% of FeSO_4_, respectively, and 1.3- and 1.6-fold in the HCD group with 0.05% and 0.1% of FeSO_4_, respectively. These results suggest that serum TC, TG, and glucose levels were remarkably elevated by iron supplementation, although there was no dose-dependency. Hepatic inflammatory parameters in both the ND and HCD groups with iron supplementation caused more severe hepatic toxicity, as AST and ALT were significantly elevated in an iron dose-dependent manner.

### 3.7. Fatty Liver Induction upon Iron Supplementation

**Figure 7:**
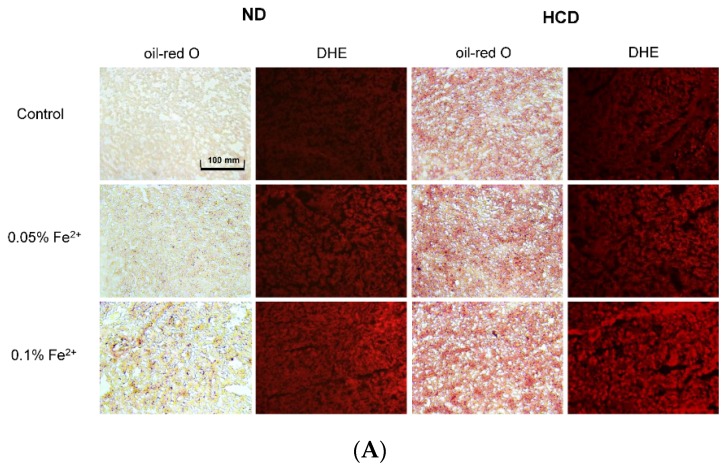

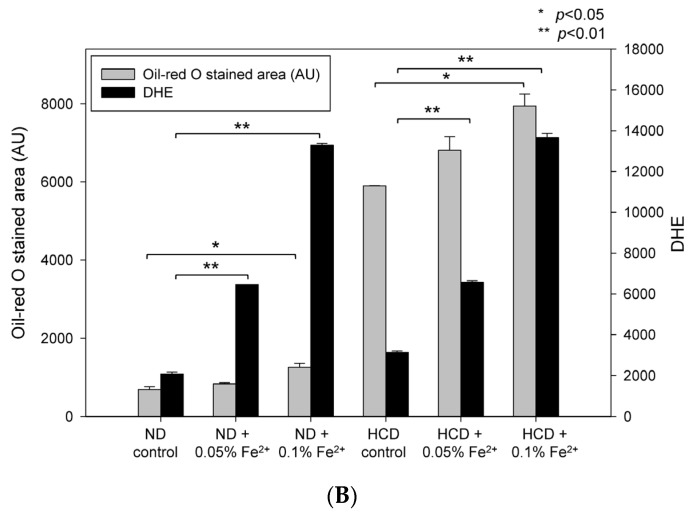
Histological analysis of hepatic tissue. (**A**) Representative sections for histological assessment of hepatic microsections after consumption of ferrous ion (0.05% and 0.1% wt/wt in diet) for 24 weeks under normal diet (ND) or high cholesterol diet (HCD). Extent of fatty liver changes was compared by oil red O staining. Production of reactive oxygen species was determined by Dihydroethidium (DHE) staining and visualized by fluorescence (Ex = 588 nm, Em = 615 nm); (**B**) Quantification of oil red O stained area and DHE stained area by computer-assisted morphometry using Image Proplus software (version 4.5.1.22; Media Cybernetics, Rockville, MD, USA). *, *p* < 0.05; **, *p* < 0.01.

In both the ND and HCD groups, fatty liver changes severely occurred upon iron consumption, as fatty streak lesions were visualized by oil-red O staining (Figure 7). In the ND group, 0.1% FeSO_4_ supplementation caused fatty liver changes without cholesterol consumption. More severe fatty liver changes occurred in the HCD group in a FeSO_4_ dose-dependent manner. The ND group showed 1.2- and 1.8-fold higher fatty liver changes upon 0.05% and 0.1% iron consumption, respectively, than the control. The HCD group showed 1.2- and 1.3-fold higher fatty liver changes upon 0.05% and 0.1% iron consumption, respectively, than the control. Reactive oxygen species (ROS) production in tissue also increased 3.1- and 6.3-fold under ND with 0.05% and 0.1% FeSO_4_, respectively, compared to the control (Figure 7B). In the HCD group, ROS production was elevated 2.0- and 4.4-fold compared to the control upon 0.05% and 0.1% FeSO_4_ consumption, respectively.

### 3.8. FeSO_4_ Consumption Reduces Embryo Production

**Figure 8:**
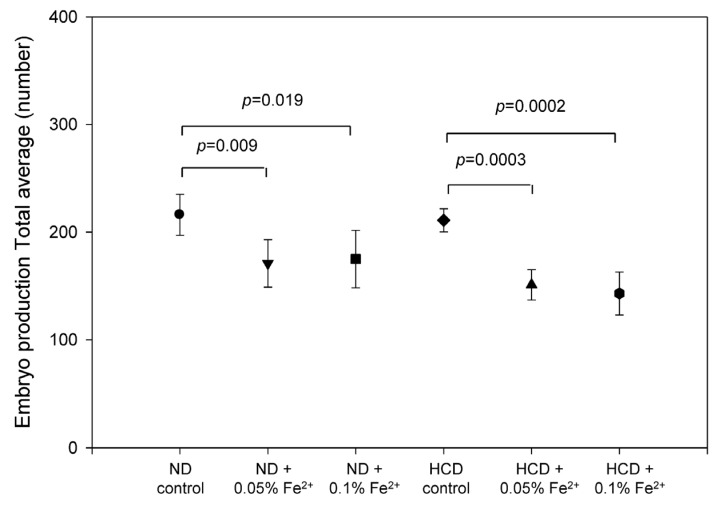
Embryo production number per mating from 18 to 24 weeks during consumption of ferrous ion (0.05% and 0.1% wt/wt in diet).

Egg production capacity was significantly reduced after 17 weeks of FeSO_4_ consumption, as shown in Figure 8. In the ND group, iron consumption resulted in a reduced egg production rate (20% lower than control) at both dosages. Upon HCD consumption, egg production rate decreased in the iron group (33% lower than control) regardless of the dosage.

### 3.9. Ovarian Tissue Was Damaged by Iron Consumption

**Figure 9:**
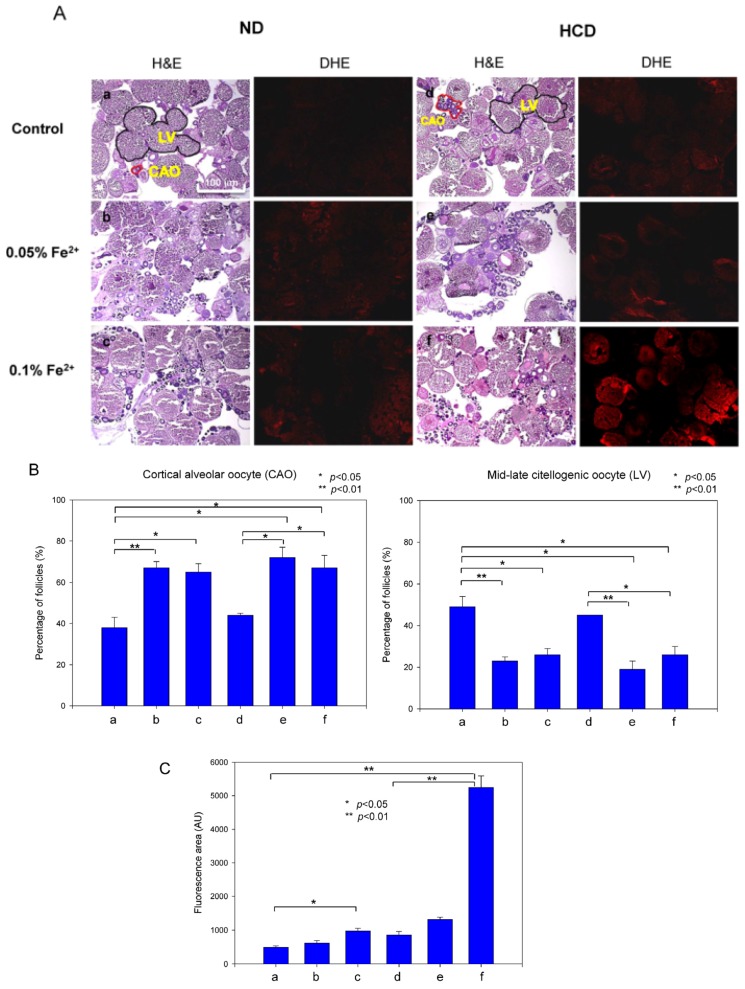
Histological analysis of ovarian tissue. (**A**) H & E staining of frozen ovarian tissues from zebrafish fed 0.05% and 0.1% Fe^2+^ under normal diet (ND) or high cholesterol diet (HCD) for 24 weeks. CAO, cortical alveolar oocytes; LV, Mid-late vitellogenic oocytes (scale bar 100 µm). Reactive oxygen species production at 48 hours post fertilization (hpf) as visualized by DHE staining using quantification of fluorescence (Ex = 588 nm, Em = 615 nm); (**B**) Graph shows the average value of CAO and LV area. a, ND control; b, ND + 0.05% Fe^2+^ (wt/wt in diet); c, ND + 0.1% Fe^2+^ (wt/wt in diet); d, HCD control; e, HCD + 0.05% Fe^2+^; f, HCD + 0.1% Fe^2+^. *, *p* < 0.05; **, *p* < 0.01; (**C**) Quantification of DHE stained area by computer-assisted morphometry using Image Proplus software (version 4.5.1.22; Media Cybernetics, Rockville, MD, USA). *, *p* < 0.05; **, *p* < 0.01.

To determine the reason for diminished embryo production, ovarian tissues were examined by H & E staining to compare folliculogenesis. In the ND group, as shown in Figure 9, follicular development was more severely impaired in the FeSO_4_ group in a dose-dependent manner. In the ND group, cortical alveolar oocytes (CAO) showed increases of 76% and 71% by 0.05% and 0.1% iron consumption, respectively. Mid-late vitellogenic oocytes (LV) showed a 47% reduction upon 0.05% and 0.1% iron consumption. In the HCD group, CAO showed increases of 63% and 52% upon 0.05% and 0.1% iron consumption, respectively. LV showed 57% and 35% reduced numbers upon 0.05% and 0.1% iron consumption, respectively, as quantified in Figure 9B. ROS production was elevated in a dose-dependent manner in the ND and HCD groups, as shown in DHE staining (Figure 9A). Notably, 0.1% FeSO_4_ and HCD consumption caused the highest ROS production—up to 6.0-fold higher than the HCD control. These results suggest that iron consumption caused ovarian tissue damage, especially upon HCD consumption, along with high oxidative stress.

## 4. Discussion

Although the detrimental effects of high iron intake on human health are well known, its toxicity on lipoproteins in terms of metabolic disease has not been determined. Furthermore, the molecular mechanism responsible for impairment of the reproduction system has not been elucidated, although iron overload and high ferritin levels are associated with polycystic ovary syndrome [29]. This study was designed to investigate the physiological effect of iron supplementation on lipoprotein metabolism and toxicity in embryonic and reproduction systems.

At the lipoprotein level, ferrous ion (final 60 and 120 µM)-treated HDL_3_ showed proteolytic degradation in 15% SDS-PAGE (Figure 1). In the co-presence of fructose and ferrous ions, protein degradation of LDL, HDL_3_, and apoA-I was significantly elevated compared with ferrous ion alone (Figure 2, Figure 3 and Figure 4). It is well known that ferrous ion reacts with hydrogen peroxide to produce powerful oxidants such as singlet oxygen and hydroxyl radicals [30]. The current results suggest that the co-existence of fructose can enforce production of pro-oxidants. These results demonstrate that ferrous ion can accelerate fructose-mediated glycation and modification of HDL and apoA-I. In HDL, ferrous ion alone caused multimerization of lipoproteins, whereas lipid-free apoA-I was less modified (Figure 3).

However, co-treatment with fructose and Fe^2+^ to apoA-I induced protein degradation (Figure 3), although it was less severe than degradation of HDL (Figure 2). This result suggests that HDL particles are more susceptible to proteolysis than lipid-free apoA-I, possibly due to the existence of matrix metalloproteinase (MMP)-9 in HDL, as previously reported [31]. Taken together, ferrous ion might contribute to the acceleration of MMP reaction to initiate proteolytic degradation.

Although oxidation extent and electromobility of LDL were not significantly changed in the presence of iron alone (Figure 4), indicating that Fe^2+^ alone did not cause severe oxidation, oxidation was more severe in the co-presence of cupric ion and ferrous ion, suggesting that there might be a synergistic effect that maximizes oxidation between heavy metals. Co-presence of Cu^2+^ and Fe^2+^ resulted in the fastest induction of oxidation (Figure 4A) and electromobility (Figure 4B).

In the presence of ferrous ion, uptake of oxLDL into macrophages (Figure 5) and induction of senescence (Figure 6) were both accelerated, suggesting that a high level of iron can exacerbate atherosclerotic and the aging process. These results are in good agreement with a previous report that Fe is involved in the oxidative modification of low-density lipoprotein (LDL), leading to the formation of Fe-rich macrophages, such as foam cells, which are responsible for atherosclerotic plaque development, progression, and subsequent vulnerability for rupture [32,33]. Moreover, co-treatment of Fe^2+^ with fructose induced increased cellular senescence in HDF cells (Figure 6). These results are in good agreement with a previous report that indicated that advanced human atherosclerotic lesions contain redox-active metal ions such as iron and copper, which promote LDL oxidation [34]. Our research group previously showed that fructose treatment could cause impairment of apoA-I as well as increase the atherogenic properties of HDL. Moreover, fructose can increase the rate of non-heme iron absorption.

After 24 weeks of iron consumption, zebrafish showed a remarkable elevation of serum cholesterol and TG levels under both ND and HCD, especially 0.05% ferrous ion supplementation. This study is the first report to demonstrate iron consumption and impairment of lipid homeostasis in a zebrafish model. Liver toxicity of iron-fed zebrafish increased in a dose-dependent manner (Figure 7). Iron overload can be toxic since the liver is the main organ for metabolism of overloaded iron. As iron excretion is very limited, accumulation of iron in the body would be higher, especially in the liver. The liver is the main organ for reduction of iron toxicity [35], causing fatty liver changes and cirrhosis.

These results suggest that 5 µg of iron, corresponding to 0.05% ferrous ion in the diet (wt/wt), is sufficient to disturb lipid and carbohydrate metabolism with embryo toxicity. Embryo production was significantly reduced upon consumption of ferrous ion under HCD. Notably, the HCD group fed 0.1% ferrous ion showed severely damaged ovarian cells and hepatic tissue along with the highest ROS production. A recent study by Eid et al. suggested that iron (considered a stress inducible intracellular second messenger) moderates toxicity and cell death inducing effects in different normal and pathological situations [36]. As it is important to study chronic iron toxicity pathologies observed in many human diseases, we hereby reported the effect of low levels of iron consumption over prolonged periods of time (24 weeks) on zebrafish. The results of our research provide a good model to study a variety of diseases such as heart disease, neurological disorders, ageing, and cancer, as these diseases are reported to be caused by chronic elevated moderate levels of iron consumption [37].

## 5. Conclusions

High consumption of iron caused hyperlipidemia, hyperglycemia, and fatty liver changes along with reduction of fertility via impairment of lipid metabolism and degradation of serum lipoproteins. These results suggest that co-consumption of fructose and iron-enriched foods, such as iron-fortified cereal, bread, and sweetened soft drinks, pose a risk of causing atherosclerosis and diabetes. Co-consumption of iron-fortified foods and fructose-enriched drink might be more dangerous to exacerbate the progression of diseases. Further studies should investigate the molecular mechanism of iron to impair lipoprotein degradation and exacerbation of glycation.

## Figures and Tables

**Table 1:** Serum parameters of zebrafish after 24 weeks of feeding with FeSO_4_ based on normal diet (ND) and high cholesterol diet (HCD).

	ND ^1^			HCD ^2^		
	Control (*n* = 100)	0.05% Fe^2+^ (*n* = 100)	0.1% Fe^2+^ (*n* = 100)	Control (*n* = 100)	0.05% Fe^2+^ (*n* = 100)	0.1% Fe^2+^ (*n* = 100)
Weight (mg)	521 ± 36 ^a,3^	465 ± 38 ^b^	465 ± 36 ^b^	578 ± 39 ^a^	435 ± 37 ^b^	470 ± 38 ^b^
Weight (mg)/height (mm)	14.5 ± 2.2 ^a^	12.2 ± 1.3 ^b^	12.7 ± 1.1 ^b^	14.7 ± 1.8 ^a^	11.7 ± 2.7 ^b^	12.2 ± 1.9 ^b^
Total cholesterol (mg/dL)	160 ± 12 ^a^	590 ± 19 ^b^	340 ±1 2 ^c^	623 ± 17 ^a^	893 ± 24 ^b^	847 ± 10 ^b^
Triacylglyceride (mg/dL)	347 ± 12 ^a^	500 ± 11 ^b^	604 ± 26 ^b^	397 ± 12 ^a^	382 ±16 ^b^	405 ±12 ^b^
Glucose (mg/dL)	188 ± 11 ^a^	455 ± 24 ^b^	369 ± 35 ^b^	206 ± 15 ^a^	253 ± 12 ^b^	278 ± 21 ^b^
AST (Karmen/mL)	264 ± 14 ^a^	390 ± 16 ^b^	539 ± 21 ^c^	351 ± 24 ^a^	441 ± 57 ^b^	551 ± 49 ^c^
ALT (Karmen/mL)	26 ± 4 ^a^	33 ± 9 ^a,b^	36 ± 12 ^b^	35 ± 3 ^a^	34 ± 10 ^a^	50 ± 2 ^b^

^1^ ND, normal diet, Tetrabit^®^: Tetrabit (47.5% crude protein, 6.5% crude fat, 2.0% crude fiber, 10.5% crude ash, containing vitamin A (29,770 IU/kg), vitamin D3 (1860 IU/kg), vitamin E (200 mg/kg), and vitamin C (137 mg/kg)). ^2^ HCD, high cholesterol diet, Tetrabit + 4% cholesterol. TC, total cholesterol; TG, triglycerides; AST, aspartate aminotransferase; ALT, alanine aminotransferase. ^3^ The mean values not sharing a common letter in the same row are significantly different between groups (*p* < 0.05).
